# Understanding bladder cancer risk: Mendelian randomization analysis of immune cell and inflammatory factor influence

**DOI:** 10.3389/fimmu.2024.1460275

**Published:** 2024-10-10

**Authors:** Hiocheng Un, Wumier Wusimanjiang, Wenhao Zhan, Xinxin Zhang, Minghao Li, Jiahao Lei, Renxuan Lin, Yuliang Zhang, Junxing Chen, Zongren Wang

**Affiliations:** ^1^ Department of Urology, The First Affiliated Hospital of Sun Yat-sen University, Guangzhou, China; ^2^ Department of Obstetrics and Gynecology, The Third Affiliated Hospital of Guangzhou Medical University, Guangzhou, China

**Keywords:** bladder cancer, immune cell phenotypes, inflammatory factors, Mendelian randomization, GWAS

## Abstract

**Introduction:**

The intricate roles of immune cells and inflammatory factors in cancer, particularly their association with the risk of bladder cancer, are not well understood.

**Methods:**

This study aimed to clarify potential causal relationships between these elements and the development of bladder cancer using genome-wide association study (GWAS) summary statistics for 731 immune cell phenotypes and 91 circulating inflammatory factors (cases=2,053; controls=287,137). The primary analytical approach was Inverse Variance Weighting (IVW), supplemented by MR-Egger regression, weighted median, and weighted mode analyses. Sensitivity analyses included Cochran Q test, MR-Egger intercept test, and Leave-one-out test.

**Results:**

The findings indicated that monocytes are positively correlated with an increased risk of bladder cancer. On the contrary, double-negative (DN) T cells, HLA DR+CD8br, and CD28 on CD28+CD45RA+CD8br T cells exhibited an inverse correlation, suggesting a possible protective effect. Furthermore, inflammatory factors IL-20, IL-22RA1, and Eotaxin were significantly associated with an increased risk of bladder cancer.

**Discussion:**

These results suggest that certain immune cell phenotypes and inflammatory factors may play a role in the development of bladder cancer and could serve as potential biomarkers for assessing tumor risk. The findings also offer new insights into the pathogenesis of bladder cancer, indicating a need for further investigation.

## 1 Introduction

According to the World Health Organization (WHO) report from 2024, bladder cancer is the 9th most common cancer globally, with over 220,000 annual deaths (1). Notably, there is significant geographical variation in age-standardized incidence rates (ASR), with the highest rates observed in Europe and North America and the lowest in Central America, as well as certain regions of Central and South Asia (1). These findings highlight the persistent global challenge of bladder cancer and the urgent need for additional research and preventive strategies. The main risk factor for bladder cancer is smoking, followed by metabolic disorders, chronic urinary tract infections, and genetic factors, all of which contribute significantly to its development (2).

Immune cells, releasing pivotal inflammatory factors as signaling molecules, are integral to the complex network that forms the human immune system, providing the primary defense against external threats. These components can induce both cancer-promoting and cancer-suppressing inflammation (3). For instance, chronic inflammation often involves immune cells such as macrophages that secrete Interleukin-6 (IL-6), which facilitates the formation of the tumor microenvironment (TME). Clinical evidence indicates that bladder cancer patients with a higher proportion of macrophages in the TME have a poorer prognosis (4, 5). Additionally, certain T cells may overproduce IL-13 and IL-17 in response to chronic inflammation, cytokines implicated in tumor growth and metastasis (3). While chronic inflammation can contribute to tumorigenesis, many immune cells also function to suppress tumor growth, activating innate and adaptive immune responses during acute inflammation to bolster anti-tumor immunity (6). In the context of adoptive cell therapy (ACT) targeting the tumor immune microenvironment, increasing CD8^+^ effector T cells within tumor-infiltrating lymphocytes (TILs) has been shown to enhance therapeutic outcomes (7). In addition, inflammatory factors play diverse roles in biological processes. Pro-inflammatory factors such as IL-1 and TNF-α can promote cancer cells’ proliferation, invasion, and metastasis, with their transcription factors being elevated in most tumors (8, 9). Clinical trials have demonstrated the success of anti-tumor treatments targeting specific inflammatory factors, suggesting their potential as predictive tumor markers (10, 11).

The relationship between immune cells, inflammatory factors, and the risk of bladder cancer is an area that has yet to be fully elucidated. Discrepancies exist within the observational research landscape, with some studies reporting a correlation between elevated levels of pro-inflammatory cytokines such as IL-17 and CXCR2 and an increased risk of bladder cancer, while others have failed to establish a significant link (12). These inconsistencies could be attributed to several factors, including potential confounders, variations in study methodologies. Moreover, the heterogeneity in study findings may reflect the limitations inherent to observational research, such as uncontrolled confounding and reverse causality, which can obscure the true nature of the associations being investigated (13). Mendelian randomization (MR) is an emerging statistical technique that addresses the limitations of traditional observational research by utilizing genetic variants as instrumental variables (IVs). This method requires fewer resources and circumvents issues of confounding and reverse causality (14). By employing two-sample MR, our study seeks to evaluate the potential causal relationships between immune cell phenotypes, inflammatory factors and bladder cancer risk. The findings may deepen our understanding of bladder cancer formation and inform the development of novel prevention and treatment strategies.

## 2 Materials and methods

### 2.1 Study design

**Figure 1:**
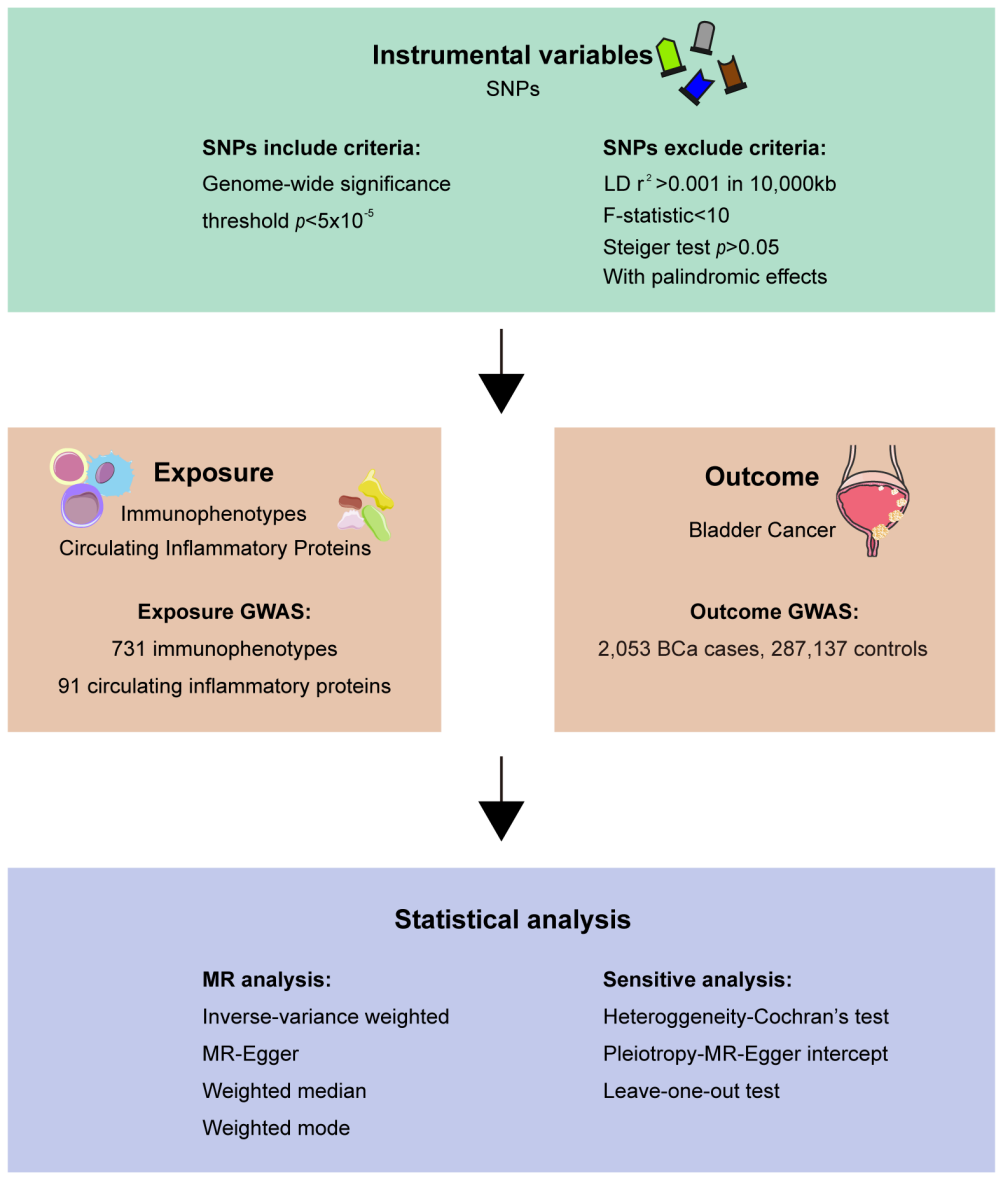
Overview of the study.

In this research, we employed single nucleotide polymorphisms (SNPs) derived from genome-wide association study (GWAS) as genetic IVs to investigate the causal associations between immune cells, inflammatory factors, and bladder cancer. A comprehensive overview of the study is depicted in **Figure 1**. We then conducted a reverse MR analysis using bladder cancer as the exposure and immune cells and inflammatory factors as the outcomes to exclude bidirectional causal associations. This study adhered to the principles outlined in the STROBE-MR (Strengthening the Reporting of Observational Studies in Epidemiology using Mendelian Randomization) framework (15, 16) (**Additional File 1** in **Supplementary Data Sheet 1**). Satisfying the subsequent three criteria: I. Correlation: The IVs exhibit a robust association with the exposure variable. II. Independence: The IVs remain unaffected by potential confounding factors. III. Exclusion restrictions: The IVs do not have a direct impact on the outcome variable, solely influencing it through the exposure variable (17).

### 2.2 Data sources

This study utilized immunological data from an extensive GWAS, which profiled 731 immune phenotypes in 3,757 individuals of European ancestry. High-density arrays were used to genotype approximately 22 million SNPs, encompassing absolute counts (AC, n = 118), relative counts (RC, n = 192), median fluorescence intensities (MFI, n = 389), and morphological parameters (MP, n = 32). Including but not limited to B cells, DCs, mature stage T cells, monocytes, myeloid cells, and TBNK (T cells, B cells, Natural Killer cells) and Treg combinations (18). Immune-wide GWAS summary data is publicly available from the GWAS Catalog (accession numbers from GCST0001391 to GCST0002121). A GWAS was conducted to investigate the genetic variants associated with 91 inflammatory cytokines. This extensive study was derived from 11 cohorts, totaling 14,824 participants of European ancestry. The study performed a genome-wide analysis of 91 quantitative trait loci (pQTL) for plasma proteins in participants and measured plasma protein concentrations using the Olink Target 96 Inflammation Immunoassay Panel (19). The study’s accession numbers range from GCST90274758 to GCST90274848 (19). GWAS summary statistics for bladder cancer are from the FinnGen Consortium R9 vision. As of May 11, 2023, the dataset contains 2,053 cases and 287,137 controls is available to the public.

### 2.3 Selection of IVs

The following are the criteria for selecting IVs for this study: I. IVs associated with each immune trait and inflammatory protein are identified with a significance threshold of 1×10^−5^, adjusted to 5×10^-5^ for bladder cancer result (20) II. The F statistic >10 is required for each immune signature and inflammatory factor for MR analysis. III. All IVs were linkage disequilibrium (LD) trimmed (r^2^ = 0.001; distance = 10,000 kb) using the R software “TwoSampleMR” package (version 0.5.8) to mitigate the impact of relevant SNPs. IV. Steiger filtering was applied to exclude invalid IVs and mitigate reverse causation (21). V. Excluded palindromic SNPs from our study. The IVs screening criteria for reverse MR analysis refer to the main analysis criteria.

### 2.4 Statistical analysis

The causal associations between 731 immune cell phenotypes, 91 circulating inflammatory factors and bladder cancer for this MR analysis were mainly estimated using the IVW method, a well-established in the MR studies landscape. The IVW method yields highly accurate estimates, operating under the assumption that all SNPs are valid instruments and that any pleiotropy present is symmetrical (22). For exposures involving more than three SNPs, the estimates for each variant were combined using the random-effects IVW method. When the exposure was instrumented by only two SNPs, the fixed-effects IVW method was used instead. To ensure that the results are stability and robustness, we also performed the following analyses: I. Supplementary Analysis using the MR-Egger regression method (this method can serve as an indicator of horizontal pleiotropy, although it provides estimates with low precision) (23), weighted median (it approach is capable of delivering reliable estimates, provided that valid IVs contribute to at least 50% of the overall weight.) (24), and weighted mode (25). II. To detect IV outliers substantially influencing causal effects, we apply Leave-one-out analysis. III. Examine the intercept values in the MR-Egger regression to assess potential directional pleiotropy (26). IV. Assessing heterogeneity using Cochran’s Q test (27).

## 3 Results

### 3.1 Selection of IVs

After applying our stringent multi-condition screening process, we identified a total of 18,728 SNPs as IVs for immune cell phenotypes and 1,465 SNPs for inflammatory factors. The F-statistic was computed to evaluate the robustness of the genetic instrument and to determine if the causal association estimates might be influenced by weak instrument bias. The F statistics for both immune cells and inflammatory factors exceeded the recommended threshold of >10, suggesting strong statistical power and unlikely weak instrument bias. Steiger’s causal analysis indicated p-values <0.05 for immune modulators significantly associated with bladder cancer, prompting the absence of reverse causation in these findings. A comprehensive summary of these results is provided in **Supplementary Tables S1, S2**.

### 3.2 Causal associations between immune cell traits, circulating inflammatory factors and bladder cancer.

**Figure 2:**
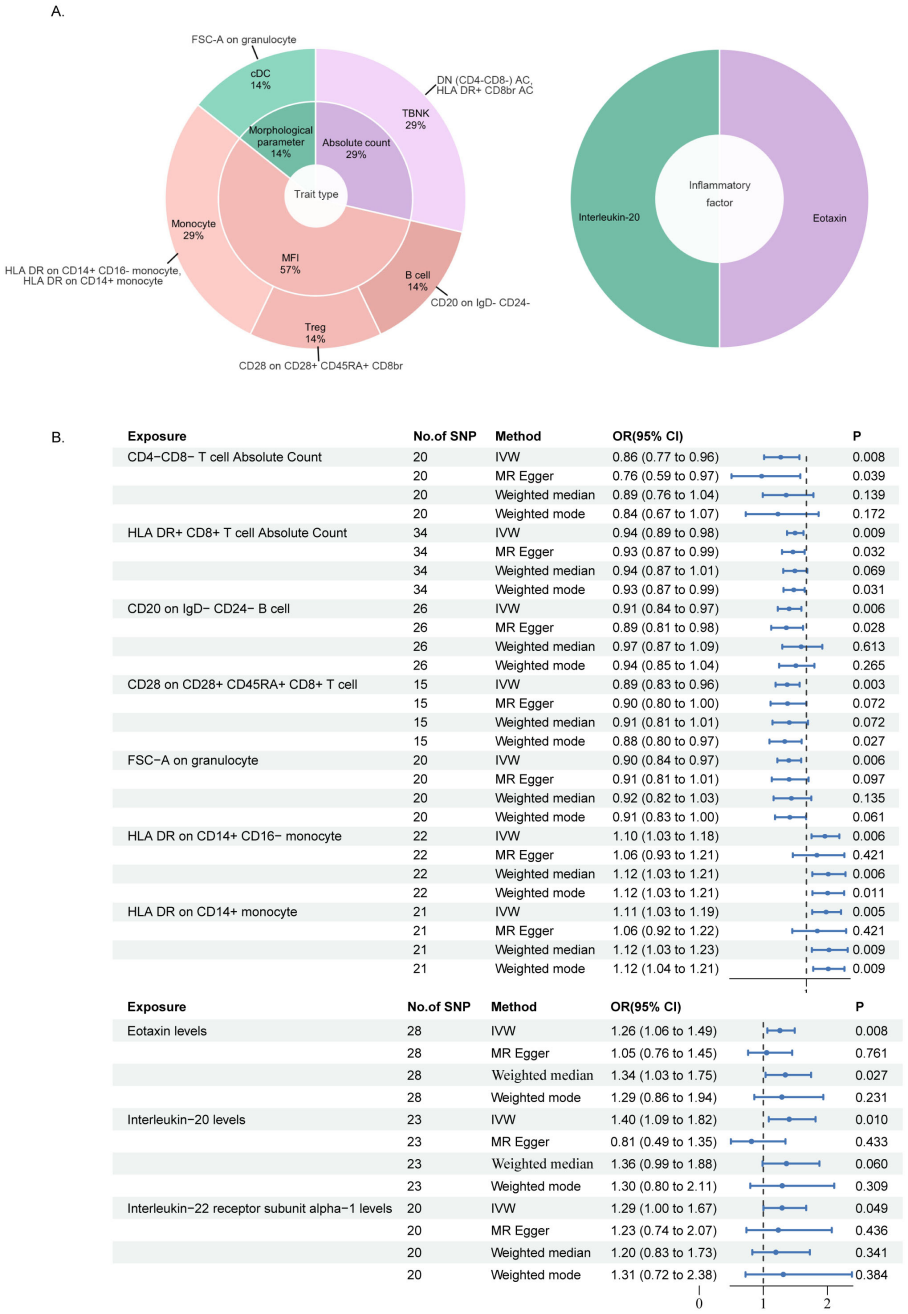
MR analysis results of immune cell phenotypes and circulating inflammatory factors in relation to bladder cancer: **(A)** is a pie chart for the results in the inverse-variance weighting (IVW) method with p<0.01. **(B)** presented the results using IVW, MR Egger, Weighted median and Weighted mode method, displaying odds ratios (OR), confidence intervals (CI), and the relevant single nucleotide polymorphisms (SNPs) associated with the study.

Utilizing the IVW method with a significance threshold of p<0.01, our study revealed the causal roles of seven immune cell phenotypes and two circulating inflammatory factors in bladder cancer development (**Figure 2A**). We identified five immunophenotypes with protective effects against bladder cancer. Including DN (CD4^-^CD8^-^) AC (TBNK panel, OR: 0.86, 95% CI 0.77-0.96, *p*=0.0083), HLA DR^+^CD8^br^ AC (TBNK panel, OR: 0.94, 95% CI 0.89-0.98, *p*=0.0087), CD20 on IgD^-^CD24^-^ B cell (B cell panel, OR: 0.91, 95% CI 0.84-0.97, *p*=0.0064), CD28 on CD28^+^CD45RA^+^CD8^br^ T cell (Treg panel, OR: 0.89, 95% CI 0.83-0.96, *p*=0.0029), and FSC-A on granulocyte (cDC panel, OR: 0.90, 95% CI 0.84-0.97, *p*=0.0060). In contrast, two immunophenotypes were identified as risk factors for bladder cancer: HLA DR on CD14^+^CD16^-^ monocyte (OR: 1.10, 95% CI 1.03-1.18, *p*=0.0060) and HLA DR on CD14^+^ monocyte (OR: 1.11, 95% CI 1.03-1.19, *p*=0.0048) which are both Monocyte panel.

Furthermore, we reveal the involvement of two circulating inflammatory factors in bladder cancer at the same screening criteria (*p*<0.01), respectively as the level of Eotaxin (CCL11, OR: 1.26, 95% CI 1.06-1.49, *p*=0.0075) and IL-20 (OR: 1.40, 95% CI 1.09-1.82, *p*=0.0097) which are all risk factors. It is noteworthy to report the identification of the protein Interleukin-22 receptor subunit alpha-1 (IL-22RA1, OR: 1.29, 95% CI 1.00-1.67, *p*=0.0490), which emerged from the results of an additional screening criterion (*p*<0.05). Serving as one of a receptor for IL-20, this protein is also observed in our findings as a potential risk factor, so we have incorporated IL-22RA1 into our subsequent discussion (**Figure 2B**).

**Figure 3:**
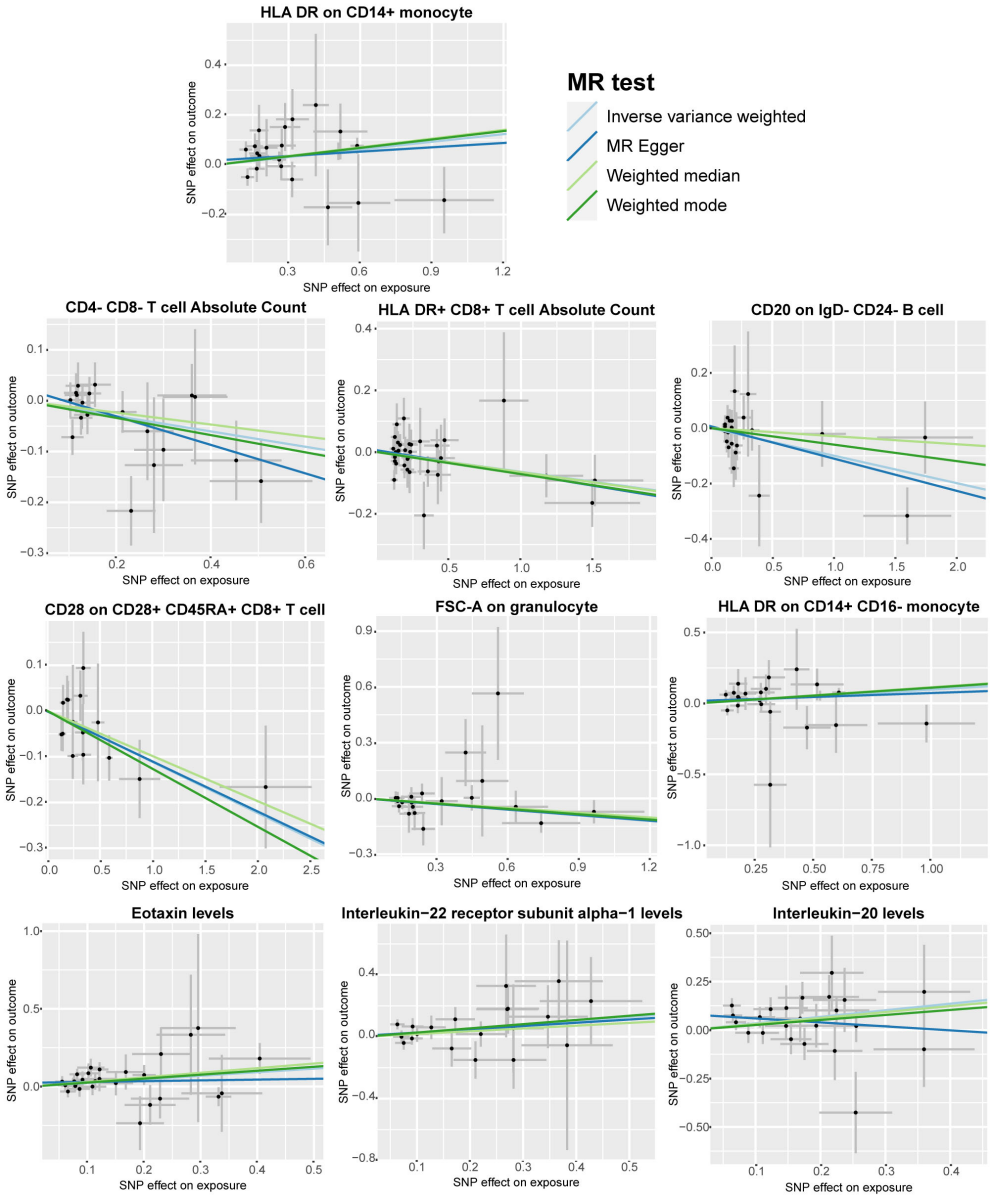
Scatter plots according to the analysis result of MR-Egger, weighted median, and weighted mode for the causal association between immune cell phenotypes and circulating inflammatory factors and bladder cancer. Illustrated the association with a focus on the relevant single nucleotide polymorphisms (SNPs) involved.

To further emphasize the robustness of these insights, results from three additional analysis methods are also presented: MR-Egger, weighted median, and weighted mode. (**Figure 3**) Although statistical significance is not achieved across all method-derived p-values, their collective directional trends remain consistently in accordance with the IVW methodology (**Supplementary Tables S3, S4**).

### 3.3 Sensitive analysis

**Figure 4:**
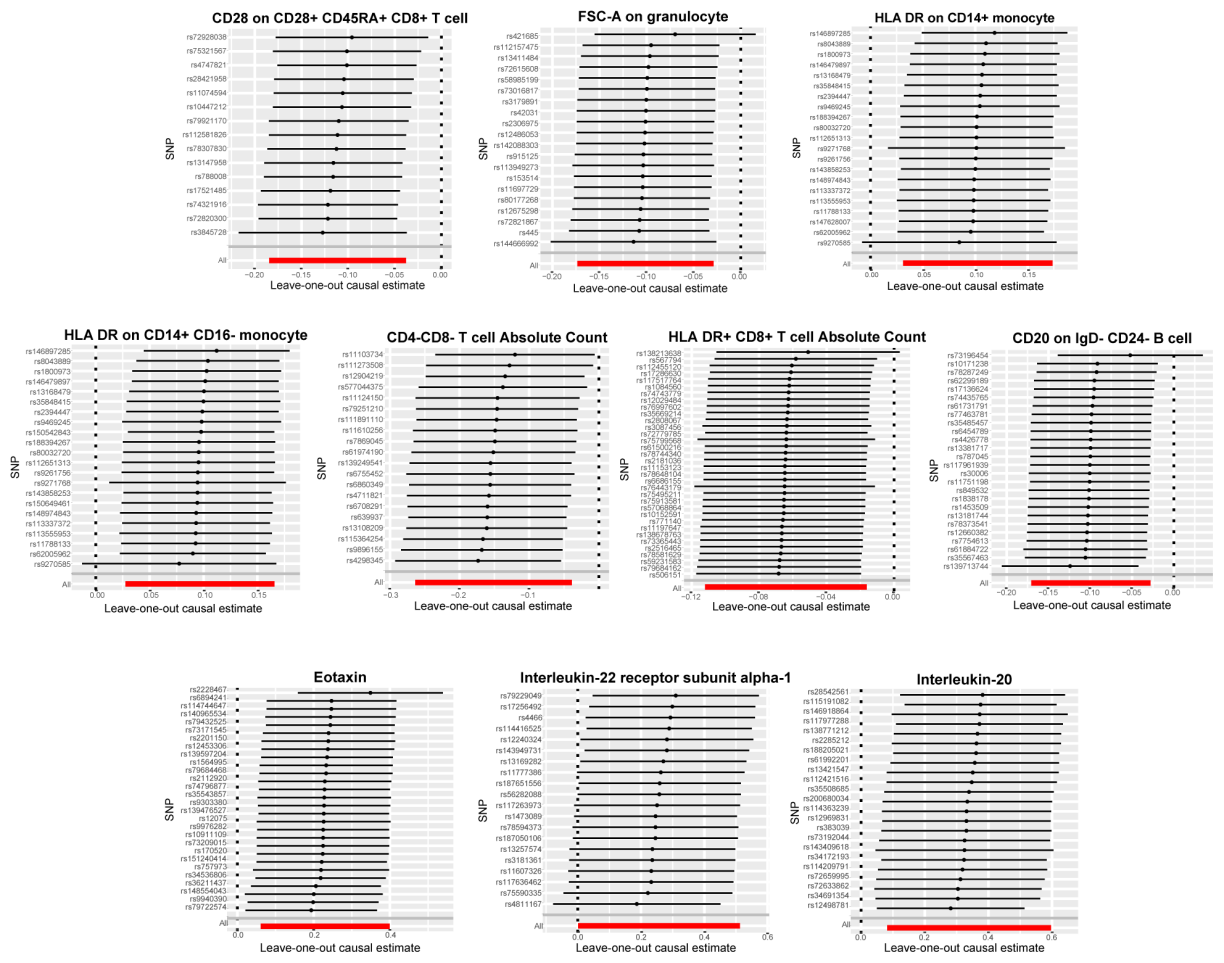
Leave-one-out plots for the causal association between immune cell phenotypes and circulating inflammatory factors and bladder cancer. Demonstrated the stability of the causal inference through a leave-one-out methodology.

Our primary analyses were complemented by a comprehensive sensitivity analysis to reinforce the validity of our findings. We utilized Cochran’s Q test (**Supplementary Tables S5, S6**) and the MR-Egger method for assessing horizontal pleiotropy (**Supplementary Tables S7, S8**), both of which yielded p-values >0.05, indicating that neither heterogeneity nor horizontal pleiotropy significantly impacted our results. Leave-one-out analyses, which involves sequentially removing each single study to examine the influence of individual data points on the overall results, are depicted in **Figure 4** and further confirming the reliability of our findings.

### 3.4 Reverse Mendelian randomization analysis

We performed reverse MR analysis on the 7 mentioned immune cells and 3 inflammatory factors. Using p<0.05 as the screening threshold, the p-values of the immune cells and inflammatory factors in the reverse MR analysis were not significant, suggesting that reverse causality is unlikely to exist. (**Supplementary Tables S9, S10**).

## 4 Discussion

To our knowledge, this is the first MR study to utilize GWAS data on immune cell phenotypes and inflammatory factors to investigate the causal roles in bladder cancer. Our results identified seven immune cell types and three inflammatory factors with significant causal associations with bladder cancer risk. Specifically, the AC of HLA DR^+^CD8^br^, CD28 on CD28^+^CD45RA^+^CD8^br^ T cell and DN AC may offer protective effects against bladder cancer. In contrast, the presence of HLA DR on CD14^+^CD16^-^ monocytes and HLA DR on CD14^+^ monocytes are associated with an increased risk of bladder cancer. Additionally, we found that the inflammatory factor IL-20 and one of its receptors, IL-22RA1, both associated with monocytes, are risk factors for bladder cancer.

The protective effects observed for HLA DR^+^CD8^br^ AC and CD28 on CD28^+^CD45RA^+^CD8^br^ T cells suggest that these immune cells may be critical in mounting an effective anti-tumor immune response. HLA DR+CD8^br^ T cells are known for their cytotoxic capabilities, which are essential for the elimination of cancer cells (28). These cells are capable of recognizing and killing tumor cells through the presentation of tumor antigens via the HLA DR complex (29) The presence of CD28 on these T cells indicates a potentially more activated state, as CD28 is a co-stimulatory molecule that, when engaged, enhances T cell proliferation and effector functions (3). The identification of DN T cells as protective factors is intriguing and warrants further exploration. DN T cells represent a small but functionally diverse population that can mediate both innate and adaptive immune responses (30). Although their exact role in bladder cancer is not fully understood, their potential to recognize a broad range of antigens without prior sensitization could make them valuable players in the early detection and response to cancer cells. Enhancing protective immune cell subsets represents a potential clinical application for bladder cancer immunotherapy or disease prevention, where strategies could involve boosting CD8+ T cell activity or expanding DN T cell populations.

Our results indicate that monocytes significantly contribute to bladder cancer progression. Classified by CD14 and CD16 markers, monocytes split into classical (CD14^+^CD16^-^), intermediate (CD14^lo^CD16^+^), and non-classical (CD14^+^CD16^+^) subtypes (31). Classical monocytes are pivotal in the TME, differentiating into TAMs that suppress T cell activity, recruit Tregs, and enhance tumor metastasis via pathways involving NF-κB and STAT3 (32). Observational studies have consistently linked a high density of TAMs to poor prognosis in bladder cancer patients (33, 34). For example, single-cell RNA sequencing has identified TAMs within distinct molecular subtypes of muscle-invasive bladder cancer (MIBC), associating their presence with adverse clinical outcomes such as disease recurrence (35). CD14^+^HLA-DR^lo/neg^ monocytes, as part of myeloid-derived suppressor cells (MDSCs), are implicated in tumor-induced immunosuppression and may modulate responses to immunotherapies (36). These findings suggest that targeting MDSCs could enhance the efficacy of immunotherapies in bladder cancer, although specific evidence for this remains limited and requires further research.

In addition, our findings highlight the role of IL-20 and IL-22RA1, a component of its receptor, in bladder cancer development. IL-20, part of the IL-10 family, signals through Janus kinase (JAK) and activates STAT3, an oncogenic transcription factor (37). STAT3’s role in inducing epithelial-mesenchymal transition (EMT) is a key mechanism in cancer invasion and metastasis, making it significant in bladder cancer progression (38). IL-22RA1 forms a functional heterodimeric receptor with IL-20RB, enabling IL-20 signaling (39). This receptor is commonly found in epithelial-origin tumors, including bladder cancer (40–42). Studies have confirmed IL-20’s presence in MIBC tissues and cells, with higher IL-20 levels correlating with increased IL-22RA1 expression in patients, associated with poorer outcomes (43, 44). This evidence underscores the ability of IL-20 and IL-22RA1 to enhance bladder cancer migration, invasion, and development.

The interactions between immune cells and inflammatory factors are complex and multifaceted. For example, monocytes can secrete IL-20 in response to various stimuli, including the presence of tumor cells or other inflammatory signals (45). This cytokine can then act in an autocrine or paracrine manner, influencing the behavior of nearby cells, including cancer cells and immune cells (46). The presence of IL-22RA1 on monocytes may enhance the responsiveness of these cells to IL-20, leading to a heightened inflammatory response (39). Furthermore, the inflammatory factors can also affect the differentiation and function of monocytes. For instance, IL-20 can promote the differentiation of monocytes into pro-tumorigenic TAMs, which can further secrete cytokines and growth factors that support tumor growth and metastasis (39). This creates a feedforward loop where inflammation drives the recruitment and activation of monocytes, which in turn produce more inflammatory factors that sustain and amplify the inflammatory response. Our research findings suggest that therapies inhibiting the IL-20/IL-22RA1 pathway may potentially mitigate the immunosuppressive effects of monocytes and improve the efficacy of the anti-tumor immune response.

It is imperative to acknowledge several limitations that may impact the interpretation and generalizability of the results. Firstly, the relatively lenient setting of the P-value range in this study, coupled with the absence of FDR correction, could potentially lead to an overestimation of the significance of the findings, necessitating further statistical analysis to ascertain their accuracy. Secondly, the study focused on a single ethnic group, which may limit the generalizability of our findings. Future research should aim to replicate these results in diverse populations to ensure the broad applicability of our conclusions. Lastly, the lack of experimental validation limits our understanding of the functional roles of the identified immune cell types and inflammatory factors in bladder cancer. *In vitro* and *in vivo* studies are needed to confirm these associations and to elucidate the underlying biological mechanisms.

In conclusion, our study provides a comprehensive analysis of the causal relationships between immune cells, inflammatory factors, and bladder cancer, offering new perspectives on the disease’s pathogenesis and potential therapeutic targets. The findings highlight the need for a deeper understanding of the immune landscape in bladder cancer and encourage the exploration of novel immunotherapeutic strategies. As our knowledge of the immune system’s role in cancer grows, so too will our ability to develop effective treatments that improve patient outcomes. however, the findings predominantly elucidate long-term biological mechanisms, and further clinical evidence is required to facilitate their application in the diagnosis and treatment of bladder cancer.

## Data Availability

The original contributions presented in the study are included in the article/**Supplementary Material**. Further inquiries can be directed to the corresponding authors.
